# Thought-based interaction: Same data, same methods, different results?

**DOI:** 10.1371/journal.pbio.3000190

**Published:** 2019-04-08

**Authors:** Reinhold Scherer

**Affiliations:** Brain-Computer Interfaces and Neural Engineering Laboratory, School of Computer Science and Electronic Engineering, University of Essex, Wivenhoe Park, Colchester, United Kingdom

## Abstract

Restoration of communication in people with complete motor paralysis—a condition called complete locked-in state (CLIS)—is one of the greatest challenges of brain-computer interface (BCI) research. New findings have recently been presented that bring us one step closer to this goal. However, the validity of the evidence has been questioned: independent reanalysis of the same data yielded significantly different results. Reasons for the failure to replicate the findings must be of a methodological nature. What is the best practice to ensure that results are stringent and conclusive and analyses replicable? Confirmation bias and the counterintuitive nature of probability may lead to an overly optimistic interpretation of new evidence. Lack of detail complicates replicability.

## Abstract 2

This Primer explores a recent debate about brain-computer interface studies, observing that confirmation bias and the counter-intuitive nature of probability may lead to an overly optimistic interpretation of scientific results; furthermore, a lack of details complicates replicability of analyses and experiments.

Brain-computer interface (BCI) technology translates brain activity pattern into messages (please refer to [1–7] for details). For persons with severe physical disability who cannot use conventional human–computer interaction devices, BCIs represent a promising strategy for maintaining or restoring communication with family and friends. Hence, when successfully implemented, BCI technology has a significant impact on the life of people; all the more for persons in a complete locked-in state (CLIS) condition.

## Interfacing brain and machine

BCIs translate patterns of brain signals, such as electroencephalogram (EEG) or functional near infrared spectroscopy (fNIRS), into messages by use of predictive statistical pattern–recognition models. Patterns are composed by features extracted from brain signals. Selection of informative features is crucial. If features do not contain useful information, pattern recognition will not work either ("garbage in, garbage out" principle). Features of different kinds are extracted from individual or multiple brain signals and combined to form a multivariate feature vector. Different cognitive and emotional processes (e.g., performing a specific mental task or attending a sensory stimulus) have different effects on feature vectors. Pattern recognition models are trained to automatically recognize these effects. Classification models assign feature vectors to a discrete category (class label), which is translated into action (e.g., move the cursor on the screen to the left). Regression models map feature vectors to real-valued quantity that is translated (e.g., to the horizontal position of the cursor on the screen). Brain signals are recorded from a user prior to BCI use, and this data is used to train the pattern recognition model. Machine-learning algorithms are applied to optimize model parameters to maximize prediction. Machine learning is also used to select informative features or to tune hyperparameters of the pattern recognition–learning algorithm. Hyperparameters are properties of the learning algorithm that cannot be learned from data. They have to be selected prior to training. Please refer to [8–10] for more details on statistical pattern recognition and machine learning.

Current BCI technology relies heavily on data-driven analysis and statistical pattern recognition. The reason is that comprehensive neuroscientific models that describe causal relationships between cognitive (emotional) processing and signal features are not available yet. Causality is essential. Correlation alone is not sufficient. Two issues that impact the performance of statistical pattern recognition methods are the nonstationary and the inherent variability of brain signals. Feature vectors extracted from data recorded on different days or from data recorded at different times on the same day may exhibit significant differences. Good practice is to regularly reapply machine-learning algorithms to new data collected from a user during BCI use and to adapt parameters accordingly. This strategy enables the brain and the machine to mutually coadapt.

Significant progress has been achieved in the field in the past decade. Translation of results into real-world applications was not so successful [11]. One reason for this may be that proof-of-concept prototypes have been mainly developed in the laboratory with healthy participants. Only a fraction of studies involve participants with disability and only very few in CLIS. Reasons for the low number of studies in persons with disability are first the limited access and second the fact that BCI experiments are time and cost intensive. Consequently, studies in persons with disability performed in real-world environments and over the period of several weeks provide very valuable insights.

In a recent *PLOS Biology* article, Chaudhary and colleagues (2017) [12] reported that communication in persons with Amyotrophic Lateral Sclerosis (ALS) in CLIS was successfully established. Authors used fNIRS, a linear support vector machine classification model, and an implicit attentional processing procedure. Typically, subjects are asked to perform specific mental tasks such as motor imagery (kinestethic mental imagination of body limb movements) or mental calculation. Instead, authors proposed the use of overlearned "automatic" questions. Participants are not required to actively imagine performing mental tasks but to effortlessly think "yes" or "no" in response to a question. Since the answers to the questions are known, the performance of correct "yes/no" recognition could be evaluated. After working with participants over several weeks, binary classification accuracies of about 70% were obtained.

Spüler [13], in a commentary published in this issue of *PLOS Biology*, questions the results presented in Chaudhary and colleagues (2017) [12]. Spüler reanalyzed fNIRS data made available by Chaudhary and colleagues (2017) but failed to reproduce the reported results. A debate about methods started. Chaudhary and colleagues’ response [14] to Spüler’s commentary is also published in this issue of *PLOS Biology*. The origin of the debate can be reduced to lack of detail. If information is missing, then it is challenging—or impossible—to reproduce analyses. Replicability of studies is a general issue in science [15–17], not only in BCI. The debate raises important questions: how to provide meaningful evidence that results are significant and valid? What is the best practice when reporting results? Due to the impact that BCIs can have on the life of persons and their environment, careful assessment and evaluation of results is sensitive.

## Model complexity, model evaluation, and hyperparameters

**Fig 1:**
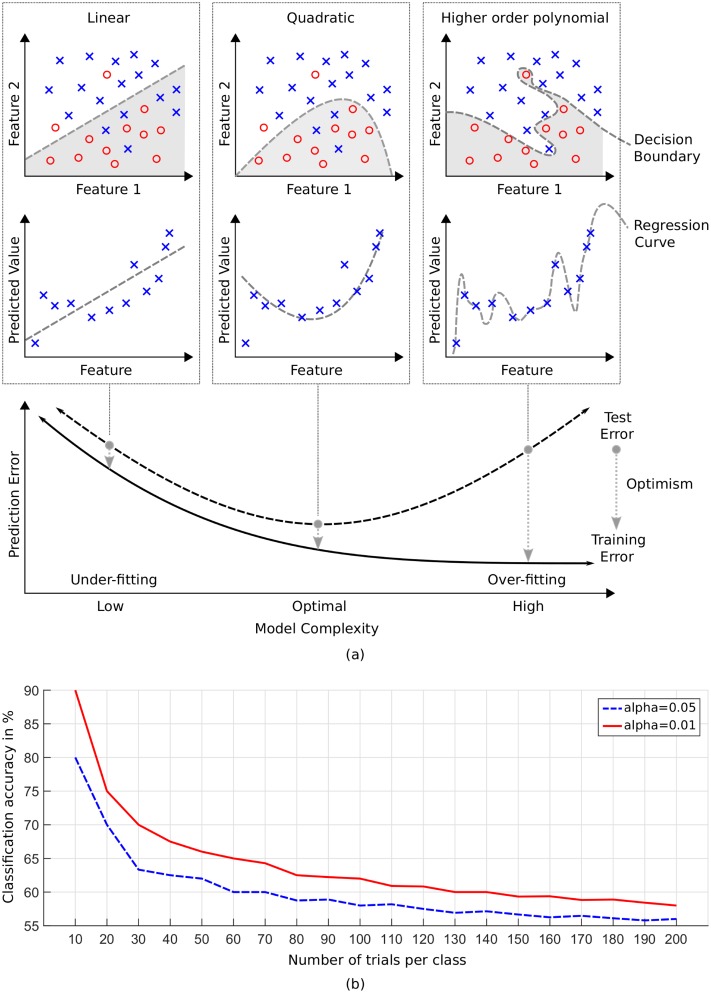
Model complexity and random accuracy. (a) The plots in the upper part depict examples of a binary classification task. The "x" and "o" symbols represent feature vectors of individual data samples of two different classes (categories). Classifier models with linear, quadratic, and higher order polynomial decision boundaries are shown. The decision boundary splits the feature space in two parts. The first characterizes the "o" (highlighted in gray) and the second the "x" pattern. The plots in the middle part illustrate examples of prediction by regression. The “x” symbols represent feature vectors. Linear, quadratic, and higher order polynomial regression curves are shown. The plot in the lower part summarizes the relationship between model complexity and the training and test error rates. Overly simple models underfit and too complex models overfit the data. Optimal models have low training and low test error. The difference between test and training error is the optimism. (b) Upper confidence limits of chance performance for a binary classification task for significance level of *α* = 0.05 (solid line) and *α* = 0.05 (dashed line). The samples for both classes are balanced. Modified from [18].

One fundamental challenge in BCI is limited data. As already mentioned, data collection is tedious and time-consuming. However, model training relies on statistics. Robust estimation of statistical measures (e.g., covariance) requires adequate amounts of data. Choice of model complexity (see Fig 1a) plays a role here. Linear models need less data than more complex nonlinear models to generalize well. Generalization refers to the capability of a pattern recognition model to perform well on new data. Models that underfit the data oversimplify the representation of the patterns (see decision boundary and regression curves in Fig 1a). These models have high bias and low variance. Bias refers to the error made due to erroneous model assumptions. Variance is the error caused by variations in the training data. Models that overfit the data memorize the pattern. They have low bias and high variance. In both cases, models will perform poorly on unseen data.

Preventing overfitting is critical when working with nonstationary and noisy brain signals. Methods such as regularization and shrinkage optimize model complexity by imposing restrictions for smoothness on the decision boundary [19]. Smoother decision boundaries mean less complex models. Regularization is a method that constrains the coefficients that describe the decision boundary. Shrinkage aims to shrink the coefficients toward zero. Cross-validation (aka jackknife) and bootstrapping techniques ensure that data used for training is different and independent from data used for testing [9]. Using the same data for training and testing obviously results in a very optimistic performance interpretation. *N*-fold cross-validation divides the available data into *N* complementary subsets (default *N* = 5, *N* = 10). The *i*-th subset is used for testing (*i* = 1…*N*). The remaining *N* − 1 subsets are used for model optimization and training. For each test set, a performance metric is computed. Generalization is estimated by calculating the average of the *N* independent performance metrics. *M*-times *N*-fold cross-validation further reduces the variance of generalization estimates by applying *N*-fold cross-validation independently to *M* permutations of the original data samples (default *M* = 5, *M* = 10). Note that it is essential that optimization and training are performed independently in each fold, i.e., a new model has to be trained for each fold. Also note that the random selection of test and training data explains small deviations in the calculated results. Brain signals are nonstationary and inherently variable. When assessing the performance, it is consequently worth considering to keep the time line of the data intact. This means that data is chronologically split into two parts. The first part is used for optimization and training by cross-validation. The second independent and temporally correct part of the data is used to evaluate the performance of trained models. This corresponds to real-world scenarios and allows most realistic estimation of generalization.

A topic that should get more attention when reporting results is hyperparameters. Different selection criteria of the hyperparameter *C* for support vector machines is likely one main reason why Chaudhary and colleagues’ and Spüler’s results are different. As stated above, hyperparameters define the behavior of learning algorithms. Different values are optimal for different patterns. Hence, it is critical to report hyperparameter values and selection criteria, including, if applicable, a description of the machine-learning algorithms used for hyperparameter selection.

## Performance metrics and randomness

The confusion matrix provides the most accurate insight into performance. The confusion matrix is a table that summarizes true positive (TP), false positive (FP), false negative (FN), and true negative (TN) recognitions. There is a number of performance metrics that can be derived from the confusion matrix [20]. Most commonly reported is the accuracy, which is the percentage share of all patterns that were correctly recognized (for two patterns, *Accuracy* = [*TP* + *TN*]/[*TP* + *FP* + *FN* + *TN*]). Essential when reporting accuracy is the question of whether or not computed accuracy is better than random. Chance performance depends on the number of patterns (classes) and their frequency of occurrence. Assume we have two patterns. If each pattern occurs the same number of times (e.g., 40 times), then the expected chance level is 50%. If we have 20 trials of pattern one and 60 trials of pattern two, then the expected chance level is 75%. That can be seen best when assuming the model, independently of the input, always outputs pattern two. High accuracy therefore does not necessarily mean good performance. When data is imbalanced, corrected accuracy is often computed by giving each pattern the same weight.

Above, only the mean value of the expected chance accuracy is considered. In order to make an informed decision on whether or not computed accuracy is better than random, the confidence interval around the expected mean chance performance has to be computed. This can be achieved analytically by using, for example, binomial statistics [18] or empirically by computing permutation tests [21]. Upper and lower boundaries of the confidence interval also depend on the chosen significance level *α* that has to be selected before the analysis (default *α* = 5%, *α* = 1%). Accuracies that exceed the upper boundary are considered to be better than random. Fig 1b shows chance level performance as function of size of the training data set. The curve shows that accuracies <80% (*α* = 5%) are likely random when only 10 trials per class are available for evaluation.

## Offline simulation versus online use

Careful performance evaluation and calculation of high offline simulation accuracy does not guarantee that the BCI user can operate the BCI online. Noise and nonstationarity—among other factors—can have adverse effects and can shift the optimal settings. Reports on users that operate BCIs in real-world environments are therefore most meaningful. To assess online performance, researchers design evaluation tasks and report to which extend BCI user succeeded in completing the tasks. However, interpretation of task performance can be challenging. For example, [22] implemented evidence accumulation to reduce incorrect selections. Users were asked to repeatedly confirm a selection before it was accepted by the BCI. To evaluate the approach, users had the task of selecting target items by row–column scanning. It turns out that some target items have a high probability of correct selection despite random BCI performance. This example illustrates that it is essential to carefully design evaluation protocols and to critically question results. Please refer to [11, 23] for more details on how to avoid common errors in BCI research.

## Clear communication is hard

BCI research is interdisciplinary and is at the intersection of natural science, social science, engineering science, and medicine. Clear and simple communication is essential. Lack of detail can lead to confusion. Confirmation bias has an influence on the interpretation of results. This is nothing new, but one has to keep it in mind. To enhance clarity of communication, reports should (i) be written in simple language; (ii) methods should be clear, precise, and include a level of detail that ensures analyzes can be replicated (sharing of source code and data); and (iii) interpretation of results should be objective and realistic—in itself a hard task.

## Abbreviations

ALS: Amyotrophic Lateral Sclerosis
BCI: brain-computer interface
CLIS: complete locked-in state
EEG: electroencephalogram
FN: false negative
fNIRS: functional near infrared spectroscopy
FP: false positive
TN: true negative
TP: true positive
